# Characterization of a New DyP-Peroxidase from the Alkaliphilic Cellulomonad, *Cellulomonas bogoriensis*

**DOI:** 10.3390/molecules24071208

**Published:** 2019-03-27

**Authors:** Mohamed H. Habib, Henriëtte J. Rozeboom, Marco W. Fraaije

**Affiliations:** 1 Molecular Enzymology, Groningen Biomolecular Sciences and Biotechnology Institute, University of Groningen, Nijenborgh 4, 9747AG Groningen, The Netherlands; m.habib@rug.nl (M.H.H.); h.j.rozeboom@rug.nl (H.J.R.); 2 Department of Microbiology and Immunology, Faculty of Pharmacy, Cairo University, Kasr El Aini 11562, Cairo, Egypt

**Keywords:** biocatalysis, protein structure, peroxidases, signal sequence, structure-inspired mutagenesis

## Abstract

DyP-type peroxidases are heme-containing enzymes that have received increasing attention over recent years with regards to their potential as biocatalysts. A novel DyP-type peroxidase (*Cbo*DyP) was discovered from the alkaliphilic cellulomonad, *Cellulomonas bogoriensis*, which could be overexpressed in *Escherichia coli*. The biochemical characterization of the recombinant enzyme showed that it is a heme-containing enzyme capable to act as a peroxidase on several dyes. With the tested substrates, the enzyme is most active at acidic pH values and is quite tolerant towards solvents. The crystal structure of *Cbo*DyP was solved which revealed atomic details of the dimeric heme-containing enzyme. A peculiar feature of *Cbo*DyP is the presence of a glutamate in the active site which in most other DyPs is an aspartate, being part of the DyP-typifying sequence motif GXXDG. The E201D *Cbo*DyP mutant was prepared and analyzed which revealed that the mutant enzyme shows a significantly higher activity on several dyes when compared with the wild-type enzyme.

## 1. Introduction

DyP-type peroxidases (DyPs) are heme-containing enzymes known for their ability to degrade dyes through their peroxidase activity. Recent studies have shown that DyPs are also involved in lignin degradation and can be used for oxidation of various compounds such as carotenoids, phenols, and aromatic sulfides. The observation that they also catalyze enantioselective oxygenation of aromatic sulfides indicates that they can even act as peroxygenases. Based on sequence features, the family of DyPs can be dissected into four subfamilies (class A, B, C, and D DyPs), according to the Peroxibase classification. Class D DyPs contain predominantly fungal peroxidases whereas the other classes mainly contain bacterial peroxidases. Members of class A typically have a Tat-signal sequence that facilitates its secretion. An example of a type A DyP is the peroxidase from *Thermobifida fusca* also known as *Tfu*DyP [1]. While this was the first reported bacterial DyP, several other DyPs have been reported in the last decade.

In this paper we identify another type A DyP from the alkaliphilic cellulomonad, *Cellulomonas bogoriensis* (*Cbo*DyP). *C. bogoriensis* was isolated from the alkaline Lake Bogoria in Kenya [2]. The predicted protein was identified by performing a sequence homology search using *Tfu*DyP. Except for typical class A sequence features such as a Tat-signal sequence and the presence of a conserved His to interact with the iron in the heme cofactor, the *Cbo*DyP sequence has an aberrant sequence in the region where normally a GXXDG motif is found. In *Cbo*DyP, the conserved aspartate is replaced by a glutamate. While this seems a mild variation, this difference from most DyPs is striking as the aspartate has been shown to be a crucial active site residue. It has been reported to play a role as a proton acceptor during the heterolytic cleavage of hydrogen peroxide. It forms a hydrogen bond with the distal solvent species and was shown to be essential for catalysis. [1,3,4] In this paper, we present a full characterization of *Cbo*DyP concerning its peroxidase activity on several dyes and its thermostability using different solvents. Furthermore, we also elucidated its crystal structure, and probed the role of the aberrant glutamate in its active site.

## 2. Results and Discussion

### 2.1. Novel DyP Enzyme Identification from C. bogoriensis

A putative DyP-encoding gene was found in the sequence genome of *C. bogoriensis* (*Cbo*DyP) after performing a BLAST search with the *Tfu*DyP sequence as a query in the NCBI (National Center for Biotechnology Information) protein database. It displays 39% sequence identity with *Tfu*DyP and also includes a Tat-signal sequence indicating that it is a class A DyP. Yet, the predicted protein sequence has an unusual sequence in the region that contains the typical DyP GXXDG motif: GXXEG. In order to study the DyP from *C. bogoriensis*, a *Cbo*DyP-encoding gene was synthesized and cloned into a pBAD-His-SUMO vector. *Cbo*DyP could be overexpressed in *E. coli* at 17 and 24 °C. The expression at both temperatures resulted in expression of soluble protein and purification yielded 84 mg L^−1^ at 17 °C and 164 mg L^−1^ at 24 °C. The *Cbo*DyP E201D mutant was also expressed in a soluble form with a yield of 68 mg L^−1^ of purified enzyme when expressed at 24 °C. To establish whether the His-tagged SUMO as fusion protein has an effect on enzyme properties, non-tagged wild-type *Cbo*DyP was prepared by using SUMO (small ubiquitin-like modifier protein) protease. Upon cleavage of the fusion protein, the peroxidase activity was found to be the same when compared with the fused version. All biochemical data reported below, except for the crystal structures, were generated using the SUMO-fused versions of both enzyme variants.

### 2.2. Spectral Properties of CboDyP

**Figure 1:**
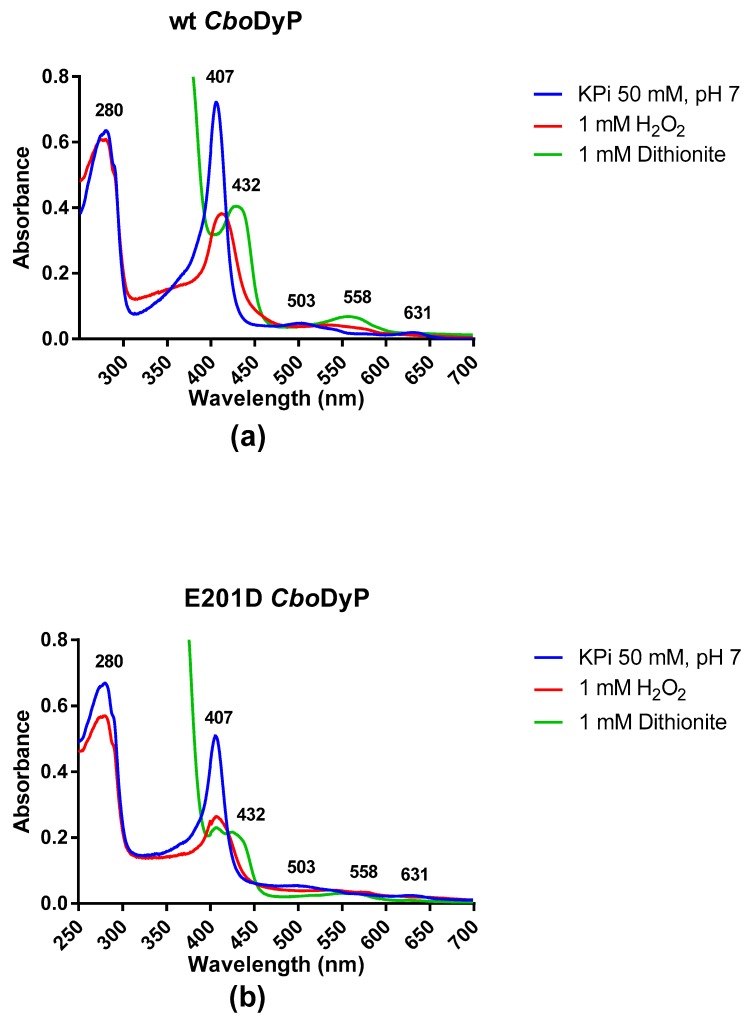
(**a**) The spectrum of wild-type CboDyP (20 µM) in KPi 50 mM, pH 7 showing a Soret band at 407 nm (blue line). The effect of the addition of sodium dithionite (green line) and the addition of hydrogen peroxide (red line) on the position and amplitude of the Soret band can be observed. (**b**) The spectrum of E201D CboDyP (20 µM) in KPi 50 mM, pH 7 showing spectral changes when adding hydrogen peroxide (red line) or dithionite (green line).

The spectrum of *Cbo*DyP shows a Soret band at 407 nm together with two less intense absorbance maxima at 503 and 631 nm (Figure 1a). The Reinheitzahl value (ratio of A407/A280) for the purified enzyme is 1.17 which is close to other values reported before for DyP peroxidases [1]. The absorbance spectrum clearly confirms that wild-type *Cbo*DyP contains a heme cofactor in the oxidized state. After having established that *Cbo*DyP is a hemoprotein, we tested the response of the enzyme to reduction using dithionite and hydrogen peroxide. Upon addition of 1 mM dithionite, the Soret band shifted to 432 nm with a reduction in the amplitude of the peak as well. The absorbance maxima at 503 shifted to 558 nm (see Figure S3a). Upon addition of 1 mM hydrogen peroxide, the Soret band shifted to 426 nm with a reduction in the amplitude of the peak. The higher wavelength peaks shifted closer together. The drastic changes in the Soret band confirm that *Cbo*DyP is a redox-active heme-containing enzyme. The absorbance spectrum of E201D *Cbo*DyP was rather similar to that of the wild-type enzyme. However, the ratio of A407/A280 is significantly lower (0.75), indicating that the enzyme may not be fully loaded with heme. Again, addition of 1 mM hydrogen peroxide or dithionite reduced the amplitude of the Soret band and had an effect on the absorbance peaks at high wavelengths (Figure 1b and Figure S3b). This indicates that the E201D mutant is also redox active. Furthermore, the spectral responses of both *Cbo*DyP variants to both redox agents confirmed that the heme had been loaded with iron. Previous work had shown that in the case of sequence-related DyPs, a high overexpression can lead to incorporation of iron deficient heme (protoporphyrin IX), resulting in inactive enzyme species [5].

### 2.3. Activity of Wild-Type (wt) and E201D CboDyP with Different Dyes

The activity of wt *Cbo*DyP and E201D *Cbo*DyP was tested in the presence of different dyes. Interestingly, as shown in Table 1, the activities of the mutant enzyme were greater for most of the dyes than the wild-type enzyme. For the anthraquinone dye Reactive Blue 19, E201D *Cbo*DyP shows the highest activity and is even more active when compared with the previously reported *Tfu*DyP [6]. Activity of both *Cbo*DyP variants with the azo dye Acid Red 14 was not very high. Also curcumin was tested but neither wild-type nor E201D *Cbo*DyP showed any activity on it. When comparing the observed activities with *Tfu*DyP, both *Cbo*DyP variants show a similar trend in activities towards different dye types, with the anthraquinone dyes being the best substrates. It is worth noting that E201D *Cbo*DyP outperforms *Tfu*DyP on most of the tested dyes. Only in one case, Disperse Blue 1, the wild-type *Cbo*DyP had the highest activity. For all other tested dyes, E201D *Cbo*DyP performed the best. The observed differences may reflect the differences in accessibility of the active site of the peroxidases.

### 2.4. pH Profile Determination for wt and E201D CboDyP

Reactive Blue 19 (RB19) was found to be one of the better substrates for wild-type *Cbo*DyP and used for further experiments. To establish the pH optimum of wild-type *Cbo*DyP and E201D *Cbo*DyP, this dye was used as test substrate. This revealed that the optimal pH for enzyme activity for the wild-type enzyme is at pH 5 while the mutant *Cbo*DyP is most active at pH 4. This may hint to a role of E201 in tuning the pH optimum to relatively high pH as would be required for an alkaliphilic bacterium. It has been observed that the pH optimum for DyPs can be substrate dependent and it is expected that *Cbo*DyP should act at a relatively high pH value. No enzyme activity for the wild-type was recorded at pH 3 and no enzyme activity for either variant was recorded at pH 7. A very interesting observation is that the activity of the mutant enzyme was almost 10-fold greater than the wild-type at a pH of 4 (see Figure S4). This could possibly be explained by the fact that the glutamate amino acid is slightly larger (extra carbon atom in the side chain) than the aspartate amino acid and therefore blocks the entrance of the active site (vide infra) thus lowering activity.

### 2.5. Steady State Kinetic Measurements for Peroxidase Activity Determination

The steady state kinetic measurements for peroxidase activity were measured for the two *Cbo*DyP variants at pH 5 using RB19 as a substrate (Figure S5). The results show that wild-type *Cbo*DyP shows less activity (*k_cat_*) than its corresponding mutant (0.22 s^−1^ vs 2.88 s^−1^). The *K_M_* values for RB19 for both enzymes are in the same range (17 µM for wild-type *Cbo*DyP and 36 µM for the E201D mutant). The *k_cat_/K_M_* values are 1.3 × 10^4^ M^−1^s^−1^ for the wild type enzyme and 8 × 10^4^ M^−1^s^−1^ for the E201D mutant. Using 100 µM RB19, the *K*_M_ values for hydrogen peroxidase were also determined: 33 µM (wild-type *Cbo*DyP) and 11 µM (E201D *Cbo*DyP), respectively (Figure S6). This again shows that the mutant enzyme is a more efficient biocatalyst.

To study the effect of the analogous mutation in other DyPs, an enzyme kinetic analysis of two other previously characterized DyPs (DyP from *Saccharomonospora viridis* [*Svi*DyP] [7] and *Tfu*DyP [1,6,8]) was performed. For both DyPs, the wild-type and the corresponding D > E mutants were prepared. Wild-type *Svi*DyP, D199E *Svi*DyP, wild-type *Tfu*DyP, and D242E *Tfu*DyP were overexpressed at a temperature of 30 °C and subsequently purified. Activities for all six enzyme variants were measured at a single substrate concentration (50 µM RB19). It was found that the D > E mutants of *Tfu*DyP and *Svi*DyP displayed a 2–5 fold lower activity when compared with their respective wild-type variants (Figure S7). This confirms that the aspartate is the optimal residue for DyPs. Yet, replacement with a glutamate retains significant activity of all tested DyPs, indicating that the DyP-identifying sequence motif can be better defined by a GXX[D/E]G motif. In fact, when we searched the sequence database with GXXEG as motif, several dozen other putative DyP sequences could be retrieved.

### 2.6. Structural Characterization of CboDyP

Dynamic light scattering analysis of the purified *Cbo*DyP indicated that the peroxidase has a hydrodynamic radius of 4.18 nm with an apparent molecular weight of 96 kDa (39% polydispersity) (Figure S9).

The crystal structure of *C. bogoriensis* dye-decolorizing peroxidase (*Cbo*Dyp) was determined to 2.4 Å resolution. The model contains eight protein molecules in the asymmetric unit (molecules A–H). The first 22 N-terminal residues of *Cbo*Dyp are not visible in electron density. Indeed, *Cbo*Dyp was predicted to contain a signal sequence for export into the periplasm encompassing the first 22 residues. Probably, the N-terminus is not ordered in the obtained crystals.

The enzyme is 54% identical to *Thermomonospora curvata* heme-containing DyP-type peroxidase [9,10] (*Tcu*Dyp, PDB code:5JXU), 43% to DtpA from *Streptomyces lividans* [11] (5MJH, 5MAP), 41% to DyP-type peroxidase (SCO3963) from *Streptomyces coelicolor* (not published) (4GT2), 39% to *Tfu*DyP [1] (5FW4), 39% to DyP-type peroxidase from *Thermobifida cellulosilytica* (not published) (4GS1), 39% to DyP-type peroxidase (SCO2276) from *S. coelicolor* (not published) (4GRC), 38% to EfeB from *E. coli* O157 [12] (3O72), and 37% to EfeB-YcdB from *E. coli* K12 (not published) (2Y4F). The enzyme has the highest homology to *Svi*DyP [7] (60% identity).

**Figure 2:**
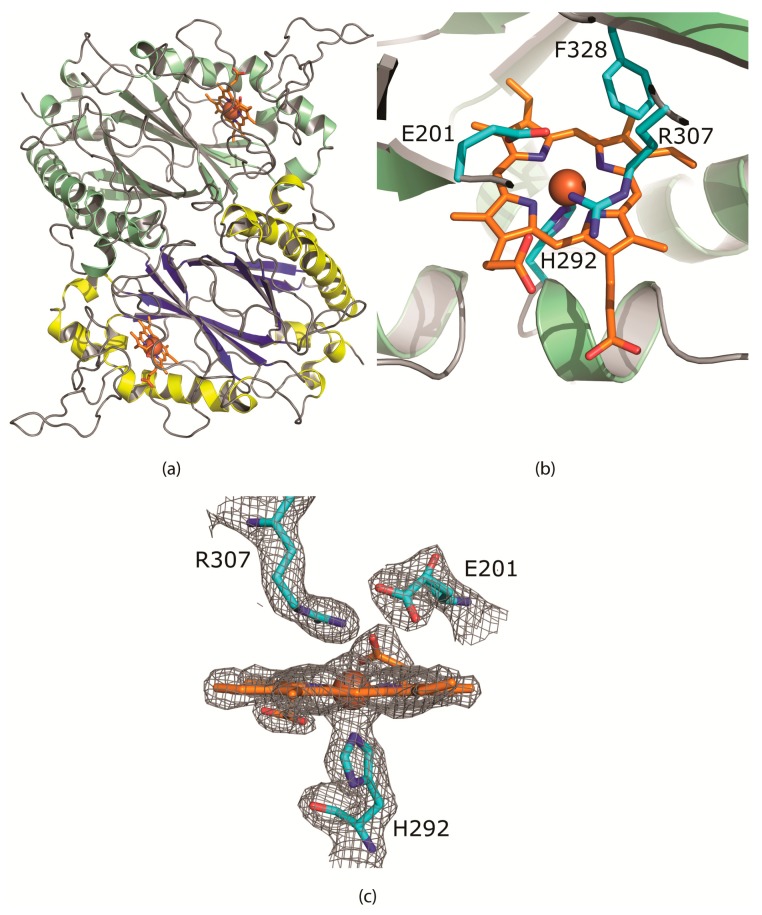
(**a**) The CboDyP dimer showing the heme and iron (brown) in the active site buried in between the alpha helices and the beta sheets and (**b**) the active site of CboDyP showing the heme and iron (brown) and the E201 residue hindering the accessibility to the active site (**c**) the σ-weighted 2mFo-DFc electron density using phases from the final model is contoured at 1.5 σ level. Contours more than 2.2 Å from any of the displayed atoms have been removed for clarity. No water or other ligands are observed near the heme cofactor.

Like other enzymes of this class, *Cbo*Dyp is a homodimer (Figure 2a). The monomer has a characteristic Dyp-type peroxidase fold containing two domains with both a ferredoxin-like fold, consisting of two four-stranded antiparallel β-sheets packed against each other [12]. The β-barrel is surrounded by 16 α-helices. The buried surface area upon dimerization of an AB-dimer is 2132 Å^2^ per monomer (Pisa server). The dimer interface is a mixture of hydrophobic and polar (1 salt bridge) interactions.

Crystal contacts are via interactions between monomers B and C (and E-H). The interactions are mainly in the loops (residues 268–281 and 294–302) (buried surface area is 460 Å^2^ per monomer). Monomers A and H (and B-G, C-F, D-E) interact via salt bridges (buried surface area is 670 Å^2^ per monomer). The crystal packing shows the presence of large solvent channels along the c-axis of the P6_2_ cell. The diameter of the pore is ~80 Å. The N-termini of all subunits are located in this channel. Dimer pairs E-F and G-H are separated by a translation of 128.1 Å along the 6-fold axis (i.e., tNCS).

The porphyrin ring of the heme cofactor is buried in a hydrophobic pocket packed between the β-strands of the N-terminal domain and two α-helices. The 2nd α-helix (residues 248–255 and 291–296) contains an excursion of 36 residues. This loop shields part of the cofactor from the solvent. The ring makes extensive interactions with predominantly hydrophobic residues; Met197, Gln199, Ala204, Ile236, Met238, Ile255, Ile293, Ala296, Phe305, Leu326, Phe328, Phe339, Val342, Leu346, Leu352, and Thr356.

The heme cofactor is ligated by the strictly conserved proximal His292 with a distance of 2.1 Å between Nε of the imidazole ring and the heme iron. The Nδ atom is hydrogen bonded to the hydroxyl of the carboxyl group of Asp350. The Asp350-carbonyl is hydrogen bonded to the backbone amides of Leu351 and Leu352. The residues on the proximal side of the porphyrin ring are well conserved in the A-class of DyP-type peroxidases.

The distal face of the porphyrin ring is ligated by Glu201 and Arg307 (Figure 2b). A glutamate residue at that position is different from all other DyP peroxidases so far known which all contain an aspartate. The extra methyl group positions the oxygens of the carboxylate group at 4.5 and 4.8 Å from the Fe of the cofactor. In 5JXU, these distances are 5.4 and 5.8 Å. The carboxylate group and the guanidine group of the arginine are also closer, 3.1 Å compared to 3.3 Å in 5JXU. The conserved Phe328 is also located on the distal face of the heme cofactor and has moved about 0.8 Å toward Glu201 by the “bulkier” Thr330, instead of Cys, Ser or Ala in the other structures. The distance between Glu201-OE2 and Phe328-CE1 has been reduced to 4.0 Å. Hence the dimensions of the distal cavity near the heme-Fe have been reduced.

The accessibility of the heme cofactor in three DyP structures (*Cbo*Dyp, *Tcu*Dyp, and DtpA) was analyzed. The Caver 3.0 analysis (see Figure S8) shows that the most important access tunnel (blue) is present at the same position in the three structures while they also all contain another access tunnel (green) which differs in each structure. The access to the internal cavity near the heme is limited by the radius of the tunnel gorge which is narrower in *Cbo*DyP (0.85 Å) compared to *Tcu*Dyp and DtpA (both 1.0 Å). This channel provides access of the hydrogen peroxide to the heme [13].

### 2.7. Thermal Stability of CboDyP

*Cbo*DyP was found to melt at a temperature of 44.75 (±0.25) °C as determined by the Thermofluor technique in 10 mM potassium phosphate buffer, pH 7. No increase in melting temperature of the enzyme was recorded for all the different solvents tested at 5% (*v*/*v*). The melting temperature dropped for all the solvents tested when shifting from 5% to 20% (*v*/*v*) concentration. The most significant finding was for acetonitrile and butanone where no melting temperature was recorded at 20% (*v*/*v*) solvent concentration as the enzyme must have already been denatured upon adding the solvent before the experiment commenced. The melting temperatures of the enzyme with and without the solvent can be seen in Figure S11.

## 3. Materials and Methods

### 3.1. Chemicals and Reagents

RB19 was supplied from Acros Organics (Pittsburgh, PA, USA). BsaI restriction enzyme was supplied from New England Biolabs (Ipswich, MA, USA). T4 DNA ligase and T4 DNA ligase buffer were supplied from Thermofisher Scientific (Waltham, MA, USA). All media components and ampicillin antibiotic were from Fischer Scientific chemicals (Pittsburgh, PA, USA). SUMO protease enzyme was provided by GECCO (Groningen, The Netherlands). InstantBlue^TM^ protein stain was purchased from Expedeon (San Diego, CA, USA). The QiaPrep Spin Miniprep kit was purchased from Qiagen (Hilden, Germany).

### 3.2. Enzyme Cloning

*Cbo*DyP was codon optimized for cloning in *E. coli* and ordered with BsaI overhangs from Thermofisher Scientific as GeneArt^TM^ Strings^TM^. The enzyme was then cloned using the NEB^®^ Goldengate Assembly Mix Protocol into a pBAD vector harboring a histag at the N-terminus followed by a SUMO protein. The pBAD-histag-SUMO-*Cbo*DyP vector was then transformed into NEB (New England Biolabs) 10β competent cells and grown overnight at 37 °C on luria bertani (LB) plates supplied with 50 µg mL^−1^ ampicillin.

The *Cbo*DyP E201D mutant, *Tfu*DyP D242E mutant and *Svi*DyP D199E mutant were constructed using the primers mentioned in Table S1 using the Quikchange mutagenesis technique. The reaction mixture contained Pfu Ultra II Hotstart PCR Master Mix 1X, 5 ng plasmid template, 0.66 µM of the primer mixture and completed to 30 µL using MilliQ water. The protocol for the PCR was: 95 °C for 4 min (initial denaturation), 33 cycles of 95 °C denaturation for 30 s, 61 °C annealing for *Cbo*DyP and 55 °C annealing for *Tfu*DyP and *Svi*DyP for 30 s, and an extension time of 3 min at 72 °C. The final extension time was 15 min at 72 °C. After the PCR was completed, DpnI was added to each of the PCR reactions to remove the non-mutated methylated plasmid and leave only the mutated clones. The samples were then treated by heating at 80 °C for 10 min to denature the DpnI. Finally, the PCR reaction products were transformed into *E. coli* NEB 10β cells and incubated on LB plates containing 50 µg mL^−1^ ampicillin, at 37 °C overnight. Colonies were then picked and used to inoculate 5 mL LB broth containing 50 µg mL^−1^ ampicillin, overnight. The overnight culture was then purified for the necessary plasmids using the Qiagen QIAprep Spin Miniprep kit adopting the manufacturer’s protocol. Finally, the purified plasmid was sent to GATC (Ebersberg, Germany) for sequencing.

### 3.3. Enzyme Expression and Purification

Plasmids from each of the three wild-type enzymes and three mutant enzymes were transformed into NEB 10β cells and inoculated onto LB agar plates containing 50 µg mL^−1^ ampicillin. An overnight pre-culture (5 mL) in LB broth was inoculated using one of the isolated colonies from each type of clone and the medium supplemented with 50 µg mL^−1^ ampicillin. The pre-culture was used to inoculate a 500 mL terrific broth (TB) medium and ampicillin was added to give 50 µg mL^−1^ as a final concentration in the medium. The culture was grown at 37 °C until an OD (optical density) of 0.6 was reached at which an amount equivalent to give a concentration of 0.02% (*v*/*v*) L-arabinose was added to induce protein expression. The flask was then transferred to an incubator operating at 24 °C and 100 rpm. After 16 h, the cultures were harvested by centrifugation at 6000× *g* for 20 min at 4 °C (Beckman Coulter, Avanti JE centrifuge, JLA 10.5 rotor). The pellet was disrupted by sonication (10 s on, 10 s off for 10 min at 70% amplitude) using a Sonics Vibra-Cell VCX130 probe sonicator. The sonicated cells were centrifuged for 30 min at 12,000 rpm to separate the cell debris from the cell-free extract. The cell free extract was filtered using Whatman FP 30/0.45 CA-S membrane syringe filters to remove remaining debris.

The cell free extract was added to gravity flow columns packed with 5 mL Ni sepharose^TM^ 6 Fast flow resin after first equilibrating the resin using 5 column volumes of Buffer A. The bound protein was washed using 5 column volumes of Buffer A followed by 5 column volumes of Buffer C. The protein was finally eluted using Buffer B until all the protein was removed from the column. The protein was then desalted using an Econo-Pac^®^ 10DG Desalting Prepacked Gravity Flow Column (BioRad) using Buffer D. The compositions of Buffers A–D are found in Supplementary Materials, Table S1. The desalted fraction was visualized by running an SDS-PAGE gel followed by staining using InstantBlue^TM^ Protein Stain to assess purity and size of the eluted protein.

### 3.4. Determination of Enzyme Concentration

The enzyme concentration of each of the six variants was determined based on the predicted molar extinction coefficient at 280 nm using the Protparam tool [14]; the extinction coefficients at 280 nm are: *Cbo*DyP = 62.575 mM^−1^ cm^−1^, *Svi*DyP = 48.595 mM^−1^ cm^−1^, and for *Tfu*DyP = 45.950 mM^−1^ cm^−1^.

### 3.5. Determination of the Activity of CboDyP with Different Dyes

A spectrum scan was done for each of Reactive Blue 19, Disperse Blue 1, and Indigotetrasulfonate using 50 µM of dye concentration to calculate the extinction coefficient at the wavelength that gave the highest absorbance. This was repeated for Acid Red 14 and copper phthalocyanine dyes but at 25 µM. Then to a plastic cuvette, 700 µL of citrate buffer was added followed by hydrogen peroxide to give a final concentration of 100 µM. The dye was then added to a final concentration of 50 µM or 25 µM depending on the dye used (see Table 1). The enzyme was finally added to give a concentration of 50 nM and the reaction followed over 90 s at the wavelength of maximum absorbance for each dye (Table 1) to determine the rate of the reaction.

### 3.6. pH Profile for CboDyP wt and CboDyP E201D Mutant

The pH profile for both the wild-type and mutant enzymes was done over a pH range starting from pH 3 to 7. To a plastic cuvette, 700 µL of citrate buffer was added followed by hydrogen peroxide to give a final concentration of 100 µM. RB19 was used as a substrate and added to a final concentration of 50 µM. Finally, the enzyme (wild-type or E201D mutant) was added to give a final concentration of 50 nM. The reaction was followed for 90 seconds at 595 nm and then the rates for both enzymes were calculated. The extinction coefficient of RB19 at 595 nm was determined by performing a spectrum scan at different wavelengths.

### 3.7. Steady-State Kinetics Measurements

The steady-state kinetic measurements for all six variants were done at pH 5 for determination of peroxidase activity. A series of increasing RB19 concentrations was prepared to be used for peroxidase activity measurement. To a plastic cuvette, 700 µL of 100 mM citrate buffer (pH 5) was added followed by hydrogen peroxide to give a final concentration of 100 µM. RB19 was used as a substrate. Finally the enzyme (six variants) was added to give a final concentration of 50 nM. The reaction was then followed for 90 s at 595 nm and then the rates for the enzymes were calculated. The extinction coefficient of RB19 at 595 nm was calculated to be equal to 10.2 mM^−1^ cm^−1^ at pH 5. The activity of *Cbo*DyP wild-type was compared to that of *Cbo*DyP E201D, *Svi*DyP wild-type, *Svi*DyP D199E, *Tfu*DyP wild-type and *Tfu*DyP D242E. This was done by measuring the decrease in absorbance at 595 nm when 50 nM of enzyme was added to a cuvette containing 50 µM of RB19, 100 µM of H_2_O_2_ and completed to volume with KPi pH 7, 100 mM buffer.

### 3.8. Spectral Properties of CboDyP Wild-Type and E201D Mutant

CboDyP wild-type and E201D mutant were analyzed to see how their spectrum scan behaved under different redox conditions. A spectrum scan of both enzymes was done in potassium phosphate buffer 50 mM at pH 7 with no additive, a second spectrum scan in the presence of 1 mM sodium dithionite, and a third spectrum scan in the presence of 1 mM hydrogen peroxide.

### 3.9. Preparation of CboDyP for X-Ray Crystallography

The SUMO protein was cleaved from the purified protein using a SUMO protease. An amount of 20 mg of protein was mixed together with 300 µL SUMO protease buffer (500 mM Tris HCl pH 8, 1.5 M NaCl, 10 mM dithiothreitol), 10 µL SUMO protease enzyme and completed to 3000 µL with milliQ water in a 5 mL Eppendorf tube. It was placed on a nutating mixer (VWR) overnight at 4 °C. The SUMO protease-protein mixture was added to 2 mL Ni-resin which was pre-equilibrated with 5 column volumes buffer A in a gravity-flow column and incubated at 4 °C on a nutating mixer for 1 h. The flow-through together with the wash using buffer B was collected. The sample was then run on an SDS-PAGE gel and protein bands were visualized by staining the gel using InstantBlue^TM^ Protein Stain (see Figure S2). The composition of buffers A and B are mentioned in Supplementary Materials, Table S2. The fraction containing the cut SUMO together with the purified protein was purified by gel filtration chromatography at 280 K using a Superdex75 10/300 GL column (GE Healthcare, Pittsburgh, PA, USA). The column was first equilibrated with 20 mM Hepes buffer, pH 7.3 and containing 150 mM NaCl. The fractions containing the enzyme were pooled and concentrated (Figure S10). Dynamic light scattering (DLS) analysis (DynaPro Nanostar, Wyatt technology, Santa Barbara, CA, USA) was carried out on the concentrated enzyme.

### 3.10. Crystallization, Data Collection, Structure Determination and Refinement

Initial sitting-drop crystallization screening was performed using a Mosquito crystallization robot (TTP Labtech) in a 96-well MRC2 plate (Molecular Dimensions) with a protein concentration of 10.6 mg mL^−1^. The screening solutions used for the experiments were PACT, Morpheus, BCS and JCSG+ (Molecular Dimensions) and Wizard Cryo (Rigaku). X-ray diffraction data were collected using an in-house MarDTB Goniostat System using Cu-Kα radiation from a Bruker MicrostarH rotating-anode generator equipped with HeliosMX mirrors. Intensity data was processed using iMosflm [15]. Several softwares were used to assess the crystal structure including constructing a Patterson map, using Phaser, Molrep, iMosflm, REFMAC5, Coot, PDB REDO, and MolProbity softwares.

After initial sitting-drop crystallization experiments were performed, hexagonal bipyramidal crystals appeared at 294 K in a solution containing 20% *v*/*v* PEG300, 0.1 M Tris buffer pH 8.5, 5% PEG8000 and 10% (*v*/*v*) glycerol (D9 in Wizard Cryo Screen). The crystals did not require supplementary cryo-protection. The *Cbo*DyP crystals belongs to the hexagonal space group P6x22 with a = b = 174.0 Å and c = 283.0 Å and has reasonable scaling statistics to 2.4 Å. Aimless suggested the presence of partial twinning (α = 0.26–0.30). Unfortunately, no reflections of the 6-fold axis were measured, therefore the space-group assignment was not possible. The presence of translational non-crystallographic symmetry (tNCS) was detected in a Patterson map [16]. A strong off-origin peak was found at a height of 41% of the origin peak. The same peak was found by Phaser [17] and Molrep [18] at fractional coordinates 0, 0, 0.453 (orthogonal coordinates 0, 0, 128, 1). The c-axis was originally determined to be 128.1 Å by iMosflm. The self-rotation function calculated by Molrep showed several extra peaks at chi = 180 °C. The space group and structure of *Cbo*DyP was determined by the molecular replacement method (MR) using Phaser [17] with a dimer of mixed model coordinates of *T. curvata* heme-containing DyP-type peroxidase [9] (PDB code: 5JXU) as a search model. Phaser did not find a solution for space group P6_x_22. However, a solution with 4 dimers in space group P6_2_ could be determined. With 8 monomers of 41.5 kDa in the asymmetric unit, the V_M_ is 3.7 Å^3^/Da [19] with a solvent content of 67%. Data collections are listed in Table S3.

The model was refined with REFMAC5 (with intensity based twin refinement) [20] and Coot [21] was used for manual rebuilding and map inspection. The twin domains H, K, L, and -K, -H, -L were refined to 0.51 and to 0.49, respectively. One TLS group per monomer was used in the last rounds of refinement. The quality of the models was analyzed with PDB-REDO [22] and MolProbity [23]. Atomic coordinates and experimental structure factor amplitudes were deposited in the Protein Data Bank (PDB) with accession code 6QZO (see Table S3).

#### Substrate Channel Calculation

The CAVER plugin for Pymol [24] was used to detect putative channels to the heme binding site of *Cbo*Dyp, *Tcu*Dyp (5JXU), and DtpA(5MJH). For calculation of the characteristics of the channels, the heme was set as a starting point for all three proteins, which were structurally aligned. Channels were calculated with the following settings: minimum probe radius: 0.8 Å; shell depth: 10 Å; shell radius: 9 Å; clustering threshold: 3.5; number of approximating balls: 12; input atoms: 20 amino acids and the hemes.

### 3.11. Thermal Stability of CboDyP

The melting temperature of *Cbo*DyP wild-type was determined using the Thermofluor technique [25]. The enzyme was diluted in potassium phosphate pH 7, 50 mM buffer to a final concentration of 1 mg mL^−1^. The fluorescence produced due to the binding of SYPRO orange dye to the exposed hydrophobic residues upon thermal protein unfolding was followed using an RT-PCR machine (CFX-Touch, BioRad). The temperature was increased from 10 °C to 99 °C using 0.5 °C increments. The temperature at the maximum of the first derivative of the observed fluorescence was taken as the apparent melting temperature. The experiment was repeated using the same concentration of enzyme and the same buffer but adding different solvents. The following solvents were tested at both 5% and 20% (*v*/*v*) concentrations: isopropanol, ethanol, methanol, acetone, acetonitrile, and butanone. Tween 80 was also tested as a solvent for the enzyme but only at 1% (*v*/*v*) final concentration.

## Figures and Tables

**Table 1:** The activities of wt *Cbo*DyP, E201D *Cbo*DyP, *Tfu*DyP [6] and hemin as a control with different dyes at pH 4.

Dye Type	Dye	λ_max_ (nm)	Conc. (µM)	*k_obs_* at pH 4 (s^−1^)			Hemin
				wt *Cbo*DyP	E201D *Cbo*DyP	*Tfu*DyP ^b^	
Anthraquinone	Reactive Blue 19	595	50	0.22 ^a^	2.88 ^a^	1.7	N.D. ^c^
Anthraquinone	Disperse Blue 1	577	50	2.26 ± 0.20	2.13 ± 0.02	10	N.D.
Azo dye	Acid Red 14	516	25	0.029 ± 0.001	0.34 ± 0.01	0.047	0.027 ± 0.038
Indigoid dye	Indigotetrasulfonate	590	50	0.051 ± 0.02	0.129 ± 0.004	0.023	0.040 ± 0.015
Phthalocyanine dye	Copper phthalocyanine-3,4′,4″,4‴-tetrasulfonic acid	616	25	-	0.137 ± 0.022	0.85	0.008 ± 0.002

^a^ These activities were measured at a pH of 5 and values represent *k_cat_*. ^b^ The activities for *Tfu*DyP are as reported in [6]. ^c^ Not determined.

## Supplementary Materials

The following are available online, Table S1: Primers for making *Cbo*DyP, *Svi*DyP and *Tfu*DyP mutants, Table S2: Buffer composition, Table S3: Data collection and refinement statistics for *Cbo*DyP, Figure S1: TatP 1.0 Server prediction of the Tat sequence for *Cbo*DyP enzyme, Figure S2: SDS-PAGE gel for His-SUMO-*Cbo*DyP protein, Figure S3: The UV–Vis spectra of wt and E201D CboDyP between 450 and 700 nm, Figure S4: pH profile of *Cbo*DyP wt and E201D mutant, Figure S5: Steady state kinetics for *Cbo*DyP and E201D mutant against RB19, Figure S6: Steady-state kinetics for CboDyP and E201D mutant against H_2_O_2_ Figure S7: Peroxidase activity of different DyPs with RB19, Figure S8: Accessibility of heme binding cavity for different DyPs, Figure S9: DLS analysis of CboDyP showing the percentage mass distribution, Figure S10: SEC elution profile of *Cbo*DyP, Figure S11: The melting temperature of *Cbo*DyP in different solvents.
