# Supplementary material for: Characterization of a New DyP-Peroxidase from the Alkaliphilic Cellulomonad, Cellulomonas bogoriensis
Source: Molecules. 2019 Mar 27;24(7):1208. doi: 10.3390/molecules24071208 (PMC6479361; doi:10.3390/molecules24071208)
Supplement: Supplementary file 1 [file molecules-24-01208-s001.pdf]

Characterization of a new DyP-peroxidase from the alkaliphilic cellulomonad, *Cellulomonas bogoriensis*

Mohamed H. Habib,[1,2] Henriëtte J. Rozeboom,[1] and Marco W. Fraaije[1*]

[1]Molecular Enzymology Group, Groningen Biomolecular Sciences and Biotechnology Institute, University of Groningen, 9747AG Groningen, The Netherlands

[2]Department of Microbiology and Immunology, Faculty of Pharmacy Cairo University, Kasr El Aini 11562, Cairo, Egypt

Supporting Information

Table of Contents

Table S1……………………………………………………………………………………………3

Table S2……………………………………………………………………………………………3

Table S3……………………………………………………………………………………………4

Figure S1…………………………………………………………………………………………...5

Figure S2…………………………………………………………………………………………...5

Figure S3…………………………………………………………………………………………...6

Figure S4…………………………………………………………………………………………...7

Figure S5…………………………………………………………………………………………...7

Figure S6………………………………………………………………………………….......7

Figure S7…………………………………………………………………………………………...8

Figure S8…………………………………………………………………………………………...9

Figure S9………………………………………………………………………………………….10

Figure S10………………………………………………………………………………………...11

Figure S11………………………………………………………………………………………...12

References………………………………………………………………………………………...13

Table S1. Primers for making the mutants of *Cbo*DyP, *Tfu*Dyp and *Svi*DyP

Mutation	Sequence
*Cbo*DyP E201D Fwd	GTTGATGGCACCGCAAATCTGGATCC
*Cbo*DyP E201D Rvs	CGGTGCCATCAACTTGACCCATCAG
*Tfu*DyP D242E Fwd	CAGATCGAAGGCACCGCCAACC
*Tfu*DyP D242E Rvs	GGTGCCTTCGATCTGCCCCATGAG
*Svi*DyP D199E Fwd	CAGTTGGAAGGTACGAGGAATCT
*Svi*DyP D199E Rvs	CGTACCTTCCAACTGGCCCATG

Table S2. Buffer Preparations

Buffer	Composition
A	50 mM potassium phosphate buffer [KPi], 0.5 M NaCl, 5% [v/v] glycerol, pH 8
B	50 mM KPi, 0.5 M NaCl, 5% [v/v] glycerol, 500 mM imidazole, pH 8
C	50 mM KPi, 0.5 M NaCl, 5% [v/v] glycerol, 5 mM imidazole, pH 8
D	50 mM KPi, 150 mM NaCl, 10% [v/v] glycerol, pH 7.5

Table S3. Data collection and refinement statistics for *Cbo*DyP. Numbers in parenthesis are for the highest resolution shell.

	*wt Cbo*Dyp
**Data collection**	
Unit cell a, c (Å)	174.0, 283.0
Resolution (Å)	58.9 - 2.40
No. of observations	1827895 (88453)
No. of unique reflections	184630 (9000)
R_{pim} (%)	11.9 (56.9)
Completeness (%)	97.9 (96.2)
Mean I/σ (I)	7.0 (1.5)
$CC_{1/2}$	0.985 (0.463)
Redundancy	9.9 (9.8)
Wilson *B* factor (Å^2)	23.2
**Refinement**	
R / Rfree (%)	24.2 / 26.6
Protein residues in A.U.	2880 (8 x 23-383)
Heme	1 per monomer
Waters	446
Geometry:	
RMSD Bond lengths (Å)	1.49
RMSD Bond angles (°)	0.007
Ramachandran favored (%)	95.30
Ramachandran outliers (%)	0.28
PDB accession code	6QZO

TatP-NN result:

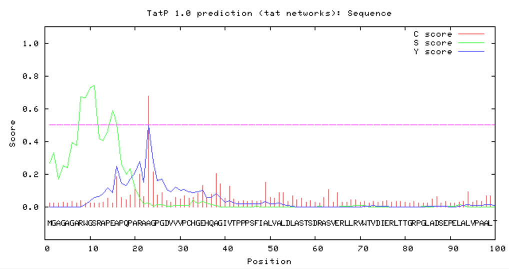


```
# data

>Sequence                 length = 100
# Measure  Position  Value  Cutoff  Tat signal peptide?
  max. C    23       0.677   0.51   YES
  max. Y    23       0.503   0.35   YES
  max. S    11       0.738   0.75   NO
  mean S     1-22    0.369   0.30   YES
  max. D     1-22    0.436   0.36   YES
# Most likely cleavage site between pos. 22 and 23: ARA-AG
# Potential Tat signal peptide but No Tat motif was found
# Used regex: RR.[FGAVML][LITMVF]
//
```

Figure S1. TatP 1.0 Server prediction of the Tat sequence for the *Cbo*DyP enzyme. The most likely cleavage site was identified by the server to be between position 22 and 23 as seen at ARA-AG.

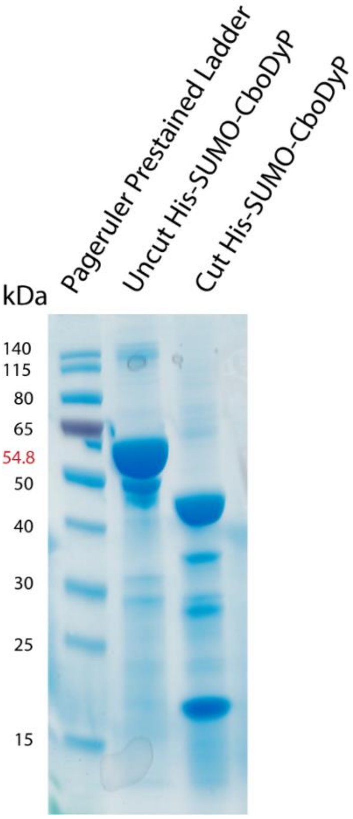

Figure S2. SDS-PAGE gel showing the uncut His-SUMO-*Cbo*DyP protein with a band at a size of ≈ 54 kDa and the *Cbo*DyP protein after cleavage of the His-SUMO using the SUMO protease with a band at a size of ≈ 41 kDa.

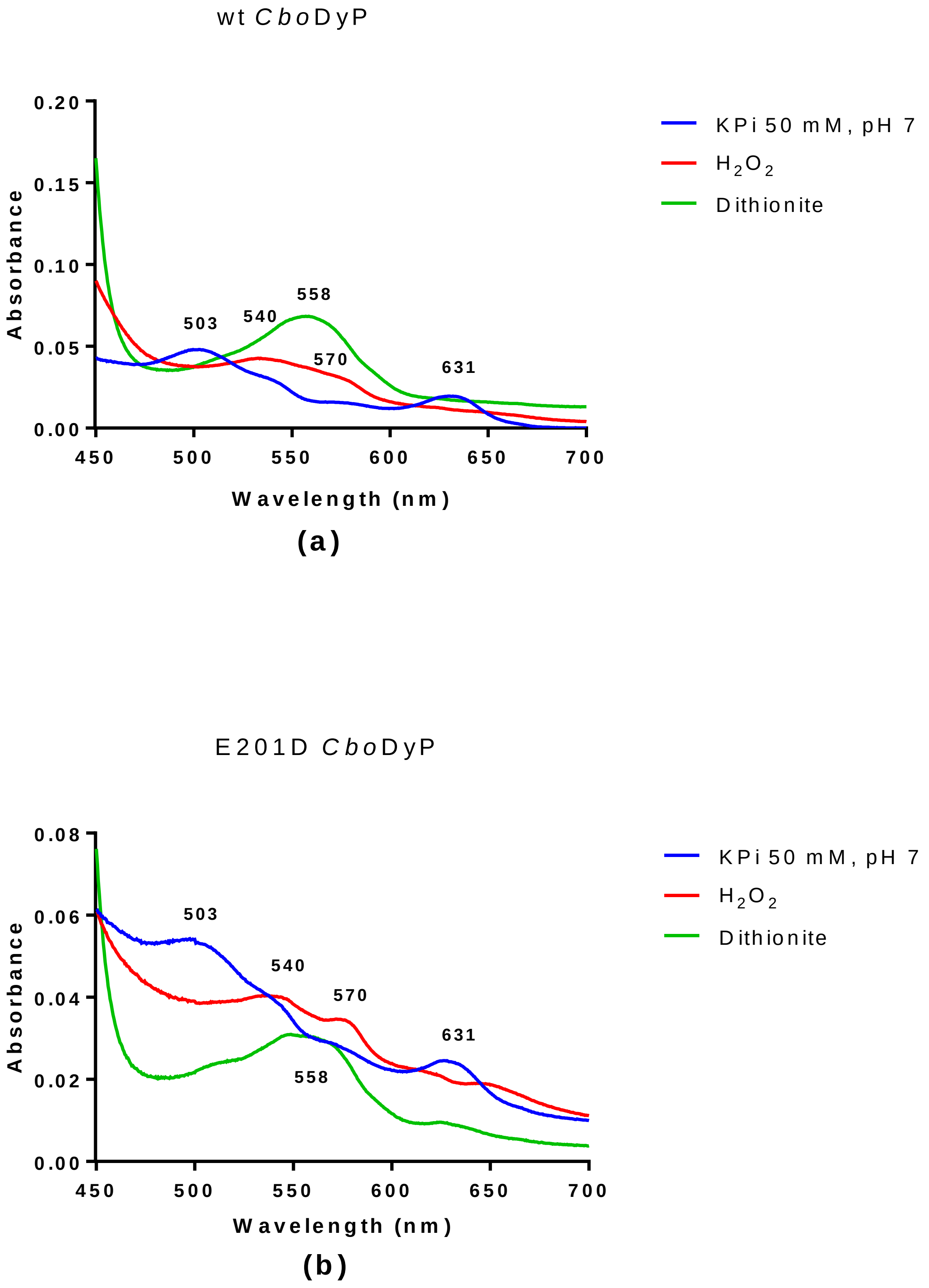

Figure S3. The UV-Vis spectra (450-700 nm) of a) wt *Cbo*DyP and b) E201D *Cbo*DyP showing the changes that occur upon addition of 1 mM hydrogen peroxide (red) or dithionite (green).

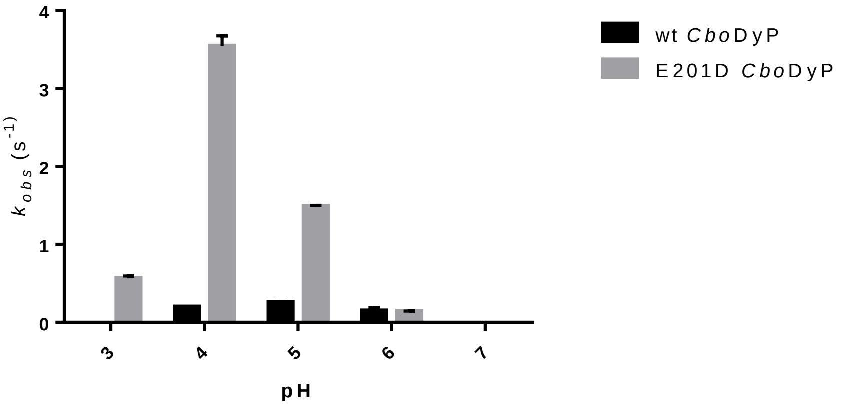

Figure S4. The pH profile of wt *Cbo*DyP versus the E201D mutant.

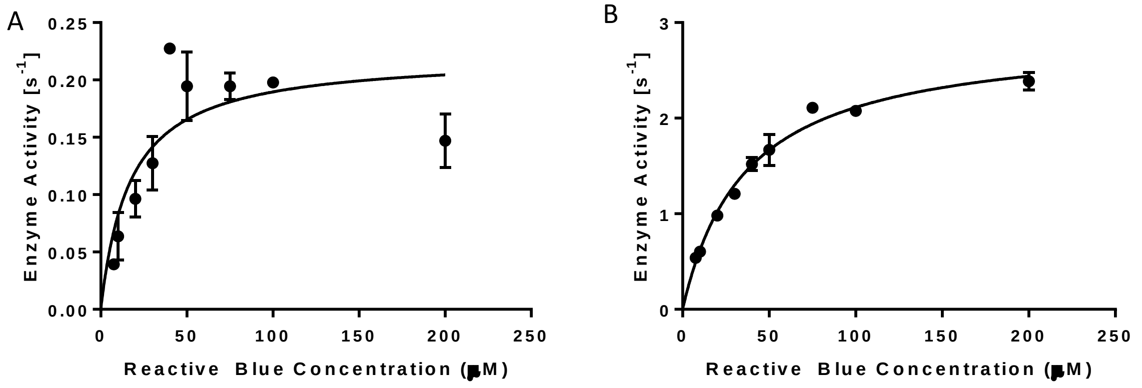

Figure S5. Steady-state kinetics for the peroxidase activity of the wild type *Cbo*DyP (A) and E201D *Cbo*DyP (B) against Reactive Blue-19 as a substrate.

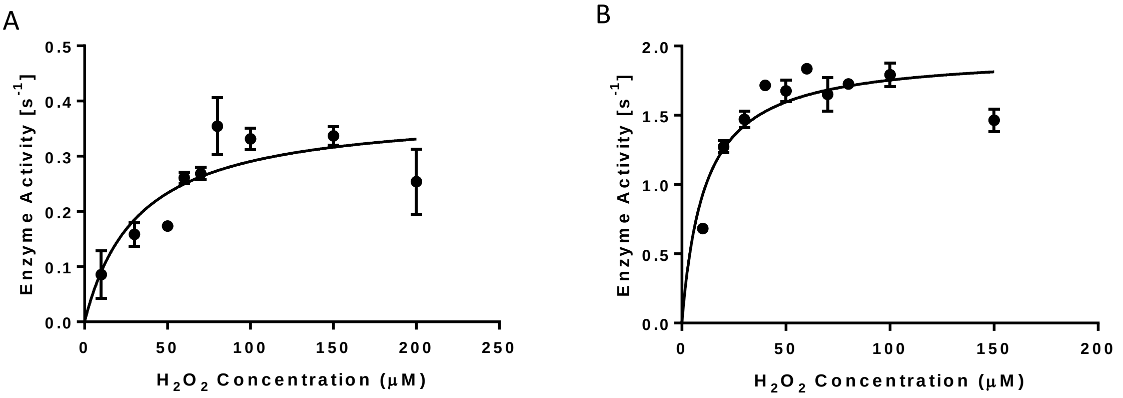

Figure S6. Steady-state kinetics for the peroxidase activity of the wild type *Cbo*DyP (A) and E201D *Cbo*DyP (B) against hydrogen peroxide as the substrate.

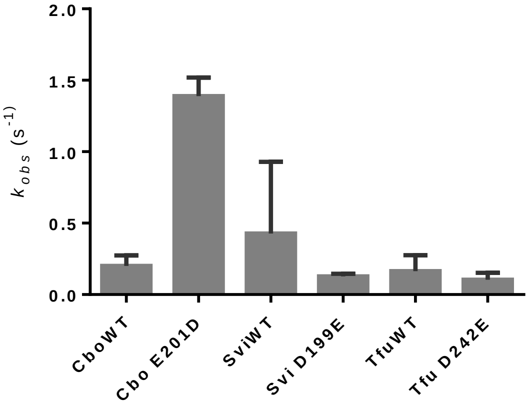

Figure S7. The peroxidase activity of the different DyPs using 50 µM RB19 as a substrate and 50 nM of enzyme in the presence of 100 µM H_2O_2.

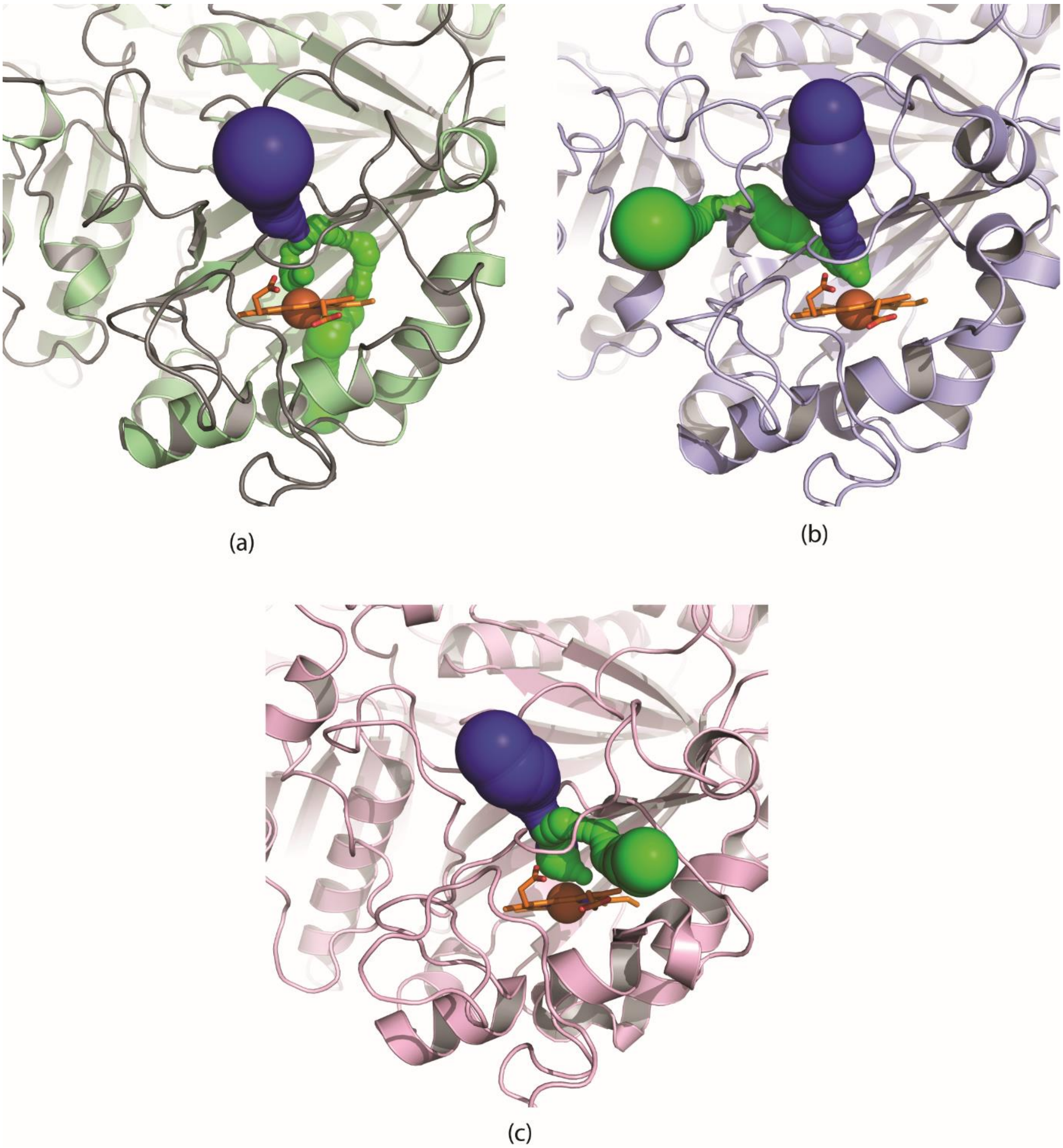

Figure S8. Accessibility of the heme binding cavity in *Cbo*Dyp (A), in *Thermomonospora curvata* heme-containing DyP-type peroxidase (B) and DtpA from *Streptomyces lividans* (C). The proteins are depicted in ribbon representation, the two most important access channels to the heme binding sites were determined with CAVER [1] and are depicted as blue and green spheres. The hemes are depicted as sticks. The green and blue channels overlap near the heme cofactor.

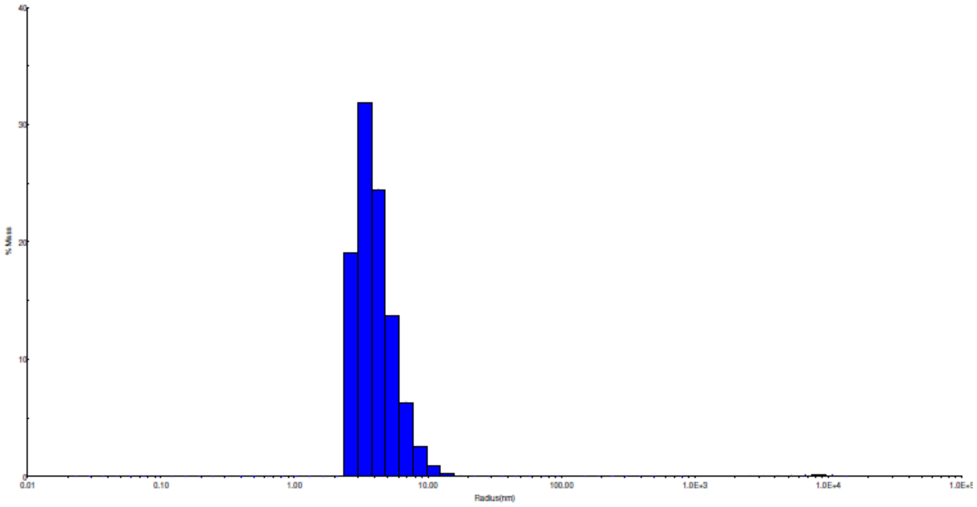

	Radius (nm)	%Pd	Mw-R (kDa)	%Intensity	%Mass
Peak 1	4.180	39.1	96	81.7	99.1
Peak 2	132.399	26.5	310430	8.7	0.1
Peak 3	305.448	19.1	2195000	3.3	0.0
Peak 4	645.334	18.1	12633200	2.0	0.1
Peak 5	5714.850	55.8	2078830000	4.3	0.7

Figure S9. DLS analysis of CboDyp showing the percentage mass distribution (from regularization analysis).

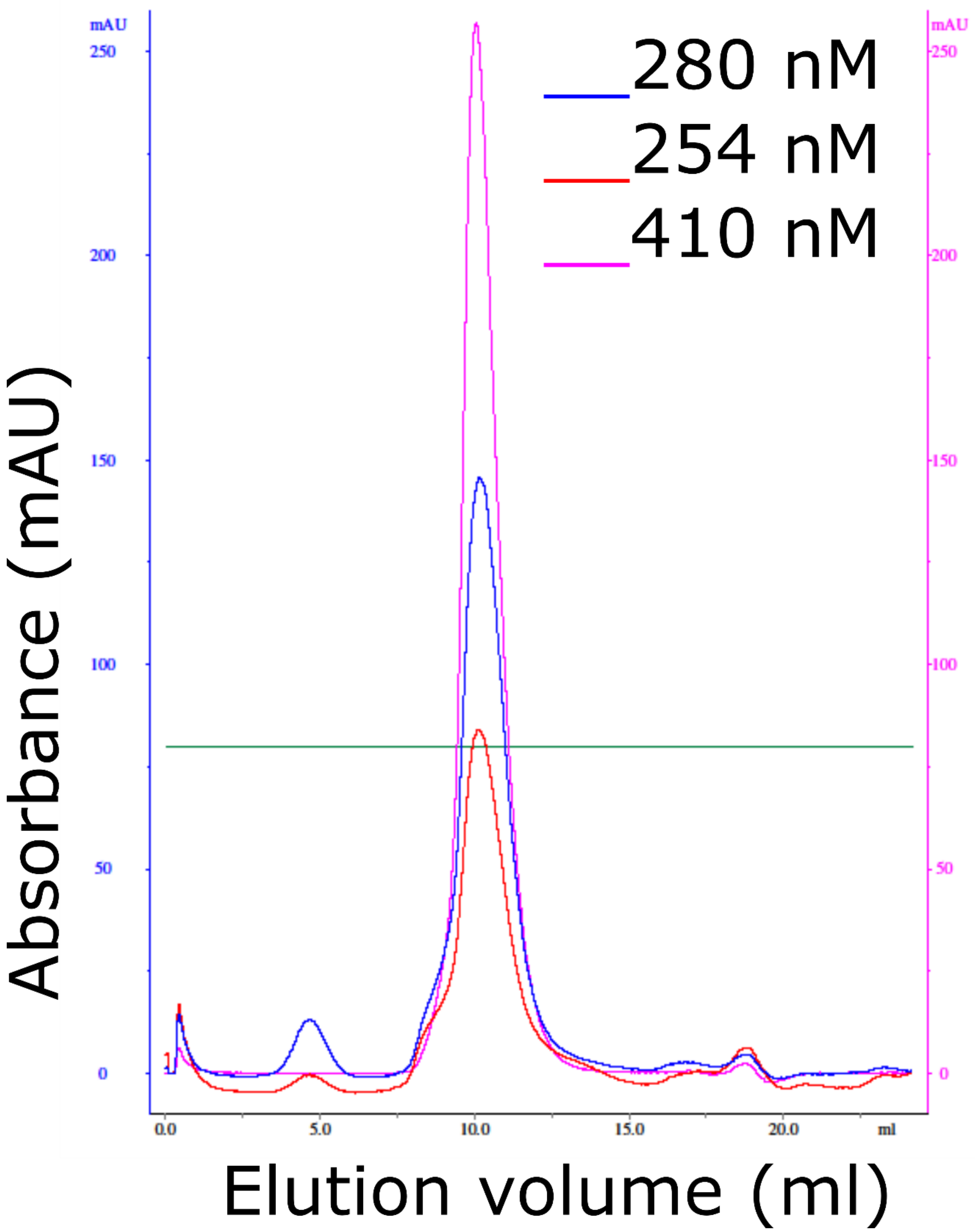

Figure S10. SEC elution profile of *Cbo*Dyp.

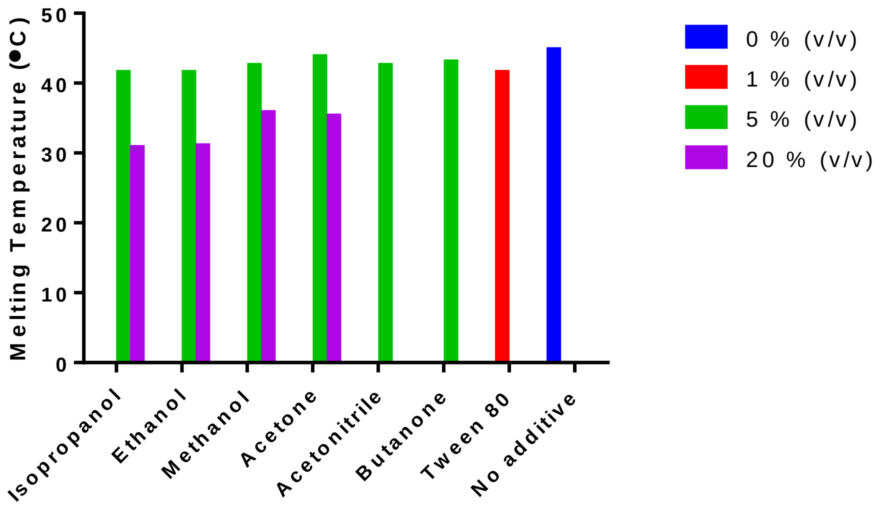

Figure S11. The melting temperature of *Cbo*DyP in the presence of different solvents at various concentrations.

References

1. Chovancova, E.; Pavelka, A.; Benes, P.; Strnad, O.; Brezovsky, J.; Kozlikova, B.; Gora, A.; Sustr, V.; Klvana, M.; Medek, P.; Biedermannova, L.; Sochor, J.; Damborsky, J. CAVER 3.0: A Tool for the Analysis of Transport Pathways in Dynamic Protein Structures. *PLOS Comput Biol* **2012**, *8* (10): e1002708.
